# Multidrug and Toxic Compound Extrusion Transporters: Ubiquitous Multifaceted Proteins in Microbes, Plants, and Their Interactions

**DOI:** 10.3390/microorganisms12122433

**Published:** 2024-11-27

**Authors:** Chiara Pastacaldi, Dario Gaudioso, Stefania Tegli

**Affiliations:** Laboratorio di Patologia Vegetale Molecolare, Dipartimento di Scienze e Tecnologie Agrarie, Alimentari Ambientali e Forestali, Università degli Studi di Firenze, Via della Lastruccia 10, 50019 Sesto Fiorentino, Firenze, Italy; dario.gaudioso@unifi.it

**Keywords:** MATE transporters, electrochemical gradient, efflux pumps, structural biology, infective diseases, antibiotic resistance, plant–pathogen interaction

## Abstract

In recent years, membrane transporters have attracted considerable interest regarding their involvement in the molecular dialogue occurring between microbes and their hosts. In particular, the multidrug and toxic compound extrusion (MATE) transporters form a family of integral membrane proteins, mainly involved in the efflux of toxic and xenobiotic compounds. They are present in all living organisms, both prokaryotes and eukaryotes, where they have a wide array of extremely different roles. In plants, MATE proteins are involved in many important physiological processes, such as plant development, as well as the active transport of several secondary metabolites. In microorganisms, they are mainly implicated in the efflux of toxic compounds and thus contribute to drug resistance. Conversely, information about the actual role of MATE transporters in the interaction between plants and microorganisms, including phytopathogens, is still limited, according to the number of publications available on this topic. Indeed, an understanding of their roles in the plant–pathogen interaction could be essential to increase the knowledge of their molecular conversation and to provide data for the design and development of innovative and sustainable anti-infective strategies to control and manage plant pathogens.

## 1. Introduction

The extrusion of toxic xenobiotic compounds is essential for any living cell. To date, seven families of multidrug transporters have been reported, including the proteobacterial antimicrobial compound efflux (PACE), the ATP-binding cassette (ABC), the major facilitator superfamily (MFS), the small multidrug resistance (SMR), the resistance nodulation cell division (RND), the AbgT (*p*-aminobenzoyl-glutamate transporter), and the multidrug and toxic compound extrusion families (MATE) [1,2].

**Figure 1:**
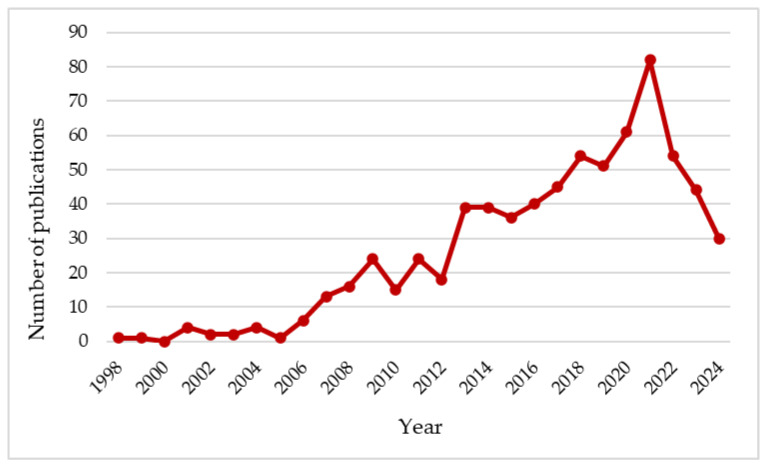
The number of publications per year reported in Scopus from 1998 to 2024, found by typing the keywords “MATE transporter” in Title or Abstract. (Data retrieved from Scopus in June 2024).

**Figure 2:**
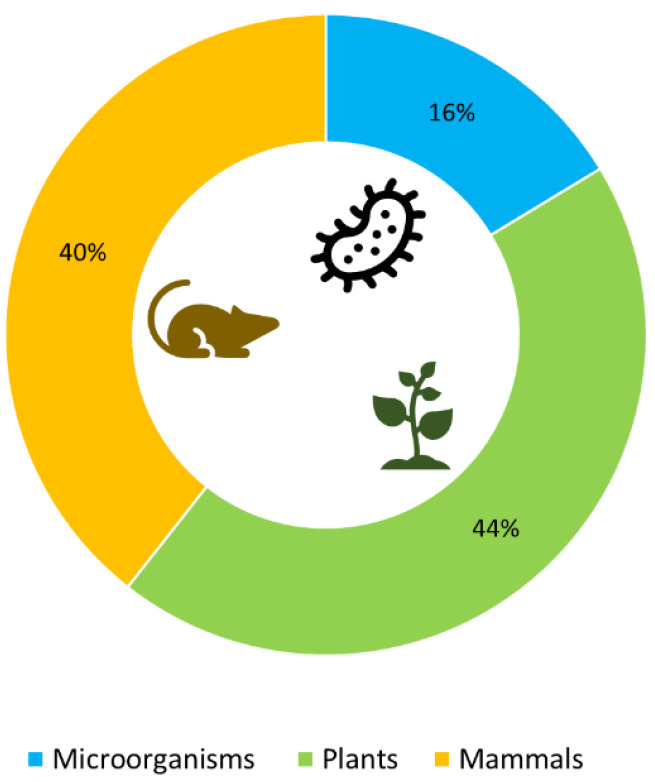
The number of papers on MATE transporters reported for different organisms from 1998 to 2024. (Data retrieved from Scopus in June 2024).

In particular, the interest in MATE-membrane transporters and their multifaced biological roles in microorganisms, plants, and mammals has been considerable in recent years, as shown in Figure 1, which shows the number of scientific papers on MATE proteins published per year, from 1998 to 2024, and indexed in Scopus. In this review, we summarize the findings from this bibliographic search, which accounts for more than 600 scientific publications. As shown in Figure 2, most of these studies were focused on plant and mammalian MATE transporters, rather than on the MATE proteins of microorganisms.

MATE transporters were first identified through sequence homology with other transporters in *Vibrio parahaemolyticus* and *Escherichia coli* [3,4]. Later, these membrane proteins were discovered to be present in Eukarya, Archaea, and Eubacteria [5]. Indeed, in 2001, the first MATE transporter of *Arabidopsis thaliana* (i.e., AtALF5) was reported and demonstrated to be involved in multidrug resistance [6]. In 2005, MATE1/SLC4781 was identified in mammals [7]. Interest in MATE transporters from animals and humans is increasing considerably, particularly due to their involvement in the secretion of a wide array of clinically important drugs [8,9].

Typically, MATE proteins have 400–550 amino acid residues, with archaeal being the smallest, while MATE transporters from yeast contain up to 700 residues [10].

The most characteristic feature of MATE transporters is their structure, consisting of 12 transmembrane helices (TMs) arranged in a unique topology with long N- and C-terminal extensions with a pseudo-two-fold symmetry [11,12]. The mammalian MATEs have an additional TM on the C-terminal of these proteins whose role remains unclear [13]. To date, more than 20 MATE transporters have been structurally characterized, mainly belonging to bacteria [14].

MATEs use a Na^+^ or H^+^ gradient across the membrane as the driving force to extrude toxic compounds, and the transport cycle is based on the transition between outward-facing and inward-facing conformation [14,15,16].

According to their amino acid sequences, three MATE subfamilies have been identified, which are NorM and DinF for prokaryotes, and eMATE for eukaryotes [4,5,17]. In some cases, bioinformatic studies have shown, through the specific analysis of the available sequences, the possibility of predicting certain functions associated with them based on their amino acid sequence [18].

The functions currently recognized for MATE membrane proteins in different living organisms are many and diverse. They include extrusion of xenobiotics and toxic compounds, accumulation of secondary metabolites, herbicide detoxification, Fe^2+^ translocation, hormone signaling, aluminum (Al) translocation, and the involvement in host–pathogen interaction [5,19,20,21,22,23,24].

Here, an overview is provided on the role of MATE transporters in microorganisms and plants, as well as in their interactions.

## 2. MATE Transporters in Microorganisms

Since the identification of NorM in *V. parahaemolyticus* and of its homologous YdhE in *E. coli* [4], many others MATE transporters have been identified in microorganisms [5,15].

Bacterial MATE proteins are mainly involved in the efflux of xenobiotics and toxic compounds, especially cationic drugs, such as ethidium bromide, fluoroquinolones (e.g., norfloxacin), and aminoglycosides [5,15]. The efflux of these compounds is coupled to the antiport of either H^+^ or Na^+^.

In *Enterobacteriaceae*, MATE transporters, such as *E. coli* MdtK and *Klebsiella pneumoniae* KetM, confer resistance to different biocides (e.g., acriflavine, benzalkonium, and ethidium) and antibiotics (e.g., norfloxacin, ciprofloxacin, and levofloxacin) [25].

In 2004, the first MATE transporter of prokaryotic plant pathogens was discovered in the Gram-negative bacterium *Erwinia amylovora*, named NorM, and demonstrated to transport several hydrophobic cationic antibiotics, such as norfloxacin, ethidium bromide, and berberine. Moreover, NorM was shown to be pivotal for *E. amylovora* fitness on Rosaceae plants, since it confers resistance to antibiotics produced by some epiphytic bacteria, especially *Pantoea agglomerans* [26].

MATE transporters are also present in fungi and yeast, where they continue to play an important role in the resistance to toxic compounds. Indeed, the MATE protein ERC1 confers *Saccharomyces cerevisiae* with resistance to ethionine, an antimetabolite and a methionine analogue and antagonist [4,5]. In the ectomycorrhizal basidiomycete *Tricholoma vaccinum*, the MATE transporter Mte1 can transport a variety of toxic compounds, such as the antibiotic hygromycin B, copper sulphate, and lithium chloride, as well as a common and broad-spectrum contact and systemic fungicide [27]. Furthermore, the Mte1 coding gene is typically induced during the symbiotic interaction of *T. vaccinum* with its host, and it has been demonstrated to mediate the secretion of the auxin plant hormone indole-3-acetic acid (IAA) [28]. Recently, some MATE transporters of the post-harvest fungal phytopathogen *Geotrichum citri-aurantii* were shown to be involved in resistance to the fungicide guazatine, and thus in the effective control of this pathogen [29]. Nevertheless, the functional and structural characterizations of the MATE transporters of these microorganisms still remain surprisingly limited.

## 3. MATE Transporters in Plants

**Figure 3:**
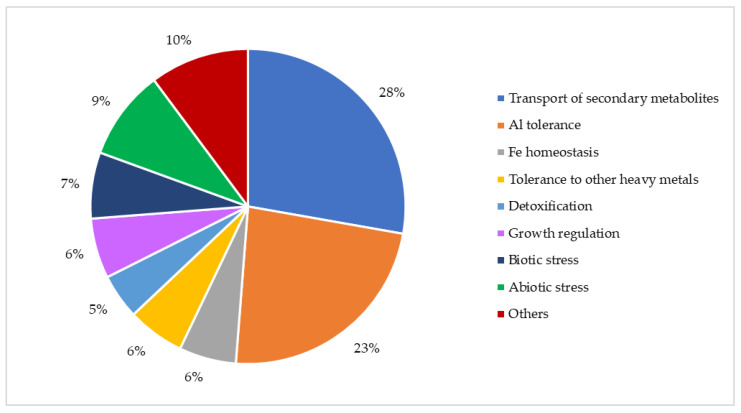
Percentage of scientific papers on the different roles of plant MATE transporters, published in peer-reviewed international journals from 1998 to 2022. The section “Others” includes single reporters on unknown and known functions (e.g., transport of CO_2_ and efflux of biological nitrification inhibitors) and general reviews on plant MATE transporters. (Data retrieved from Scopus in June 2024).

The MATE transporters of plants are also of considerable interest, generally for their role in the transport and/or extrusion of metabolites involved in many fundamental physiological and biochemical processes, such as growth and development, detoxification, active defense, communication, and maintenance of cellular homeostasis [30,31] (Figure 3). These plant MATEs are a typical preferential route for the transport of a wide array of molecules from the cytoplasm to the apoplast or into the vacuoles [32]. The role of plant MATE transporters in response to the main plant stressors is already fully recognized, although most of their molecular mechanisms need to be unveiled [31]. Moreover, the pivotal importance of MATE proteins in plants is also suggested by their high number for each species, as shown for *A. thaliana*, *Glycine max*, and *Nicotiana tabacum*, with 56, 117, and 138 MATE proteins coded in their genomes, respectively [33,34,35]. Up to now, studies have been carried out on MATE transporters of plants from different botanical families, some phylogenetically distant from each other, such as *Oryza sativa* for Gramineae [36], *Malus domestica* for Rosaceae [37], *Lotus japonicus* for Leguminosae [38], *Populus trichocarpa* for Populaceae [39], and *Pinus radiata* for Pinaceae [40].

One of the main known functions of plant MATEs is associated with the transport of secondary metabolites, such as flavonoids and alkaloids. Plant secondary metabolites are pivotal molecules in plant defenses against pathogens and herbivores, and they are involved in plant protection against UV light [41,42]. However, some of these compounds can be toxic for the plant cells when they are mislocated or in high concentrations. Thus, they are usually translocated and accumulated in specific compartments, such as vacuole and apoplast [41,42]. The transport of secondary metabolites can be intercellular, intracellular, and intratissue. The intercellular and intracellular transport is mainly mediated by the ABC and MATE transporters [42]. For instance, the first MATE protein discovered to transport flavonoid was the TT12 protein of *A. thaliana*, which mediates the accumulation of glycosylated flavan-3-ol monomers in the vacuole of the seed-coat endothelium [43,44]. Since flavonoids are also involved in organ pigmentation, mutations in the *tt12* gene sequence have been reported to alter the characteristic brown coloration of *A. thaliana* seeds [43]. The FFT/DTX35 transporter of *A. thaliana* is also involved in anthocyanin accumulation in the seed coat [45]. Similarly, the AM1 and AM3 proteins of *Vitis vinifera* have been shown to mediate the vacuolar transport of anthocyanins, which are involved in the red, blue, and purple pigmentation of berry skin [46]. Recently, the VvMATE38 has been also suggested to mediate anthocyanin transport in berry skin of *V. vinifera* [47]. Similarly, the transient overexpression of *PbrMATE9* was shown to be involved in anthocyanin accumulation in pear peel [48]. The transport of proanthocyanins and anthocyanins mediated by MATE proteins has been also reported for many others plant species, such as MTP77 of *Solanum lycopersicum*, MtMATE1 and MtMATE2 of *Medicago truncatula*, and MdMATE1 and MdMATE2 of *M. domestica* [35,41].

Moreover, other studies have reported the fundamental role of MATE proteins in the transport of alkaloids, especially in nicotine efflux toward the vacuole in *N. tabacum*. Indeed, the NtJAT1 (jasmonate-inducible alkaloid transporter 1) and NtJAT2 proteins mediate the transport of nicotine mainly in leaf vacuoles [49,50]. Another two MATE transporters of *N. tabacum*, NtMATE1 and NtMATE2, have been shown to transport nicotine into root vacuoles, and these results have also been confirmed by the heterologous expression of NtMATE1 and NtMATE2 in yeasts [24,51].

Another important and well-studied function of plant MATEs is related to Al tolerance. The accumulation of Al ions (Al^3+^) in acidic soils poses sever problems to crop productivity. Indeed, high concentrations of Al^3+^ inhibit primary root elongation, leading to a reduced plant growth [52]. Al^3+^ detoxification occurs mainly through the extrusion of citrate from the roots, which chelates Al ions in the rhizosphere [32,41]. The extrusion of citrate is mediated by MATE transporters, as demonstrated for AtMATE in *A. thaliana*, PtrMATE1 and PtrMATE2 in *P. trichocarpa*, AhFRDL1 in *Arachis hypogea,* and GmMATE13 in *G. max* [39,53,54,55]. Al tolerance can be also achieved in transgenic plants overexpressing heterologous genes coding for specific MATE transporters, as recently demonstrated by Ribeiro et al. [56]. Indeed, sugarcane transgenic plants constitutively expressing the *Sorghum bicolor* MATE gene *SbMATE* exhibited enhanced Al tolerance.

Plant MATE transporters are also involved in the regulation of iron homeostasis. Iron is a fundamental micronutrient in many physiological processes, such as respiration or photosynthesis [57]. The FRD3 transporter is reported to help the solubilization of iron in an Fe–citrate complex in *A. thaliana* and soybean, allowing its distribution throughout the whole plant [58,59]. Similarly, OsFRDL1 transports citrate, ensuring Fe translocation from roots to shoots in rice [60]. In the leguminous plant *L. japonicus*, LjMATE is induced during the formation of the symbiotic nodule by *Mesorhizobium loti* to aid iron translocation and, thus, to maintain cellular homeostasis [38]. However, not all MATE proteins carry citrate in Fe^2+^-regulation and Al-tolerance mechanisms [61]. Upadhyay et al. [41] reported three MATE transporters that are linked to Al tolerance but independent of citrate production. Additionally, the citrate-transporter MATEs now seem to be identifiable by bioinformatics analysis according to their 50-amino-acid citrate exuding motif [41].

Another important function revealed for plant MATE transporters is associated with their detoxification ability, mainly for ethidium bromide, allelochemicals, alkaloids, cadmium, lead, and cooper [62,63,64,65]. Moreover, plant MATEs are also implicated in tolerance to salt stress, a worldwide challenge and another emerging problem for the Mediterranean agroecosystems [66]. The overexpression of several *DTX/MATE* genes from *Gossypium hirsutum* into *A. thaliana* demonstrated their role in enhancing the tolerance of this model plant against multiple abiotic stresses, such as salinity, drought, and cold stress [67]. The *AtDTX1* gene of *A. thaliana* was the first gene reported to code for a transporter for multidrug resistance, able to extrude both antibiotics and cadmium [62]. The AtDTX3 transporter was also shown to have a potential role in bioremediation [68]. Another MATE protein of *A. thaliana* was demonstrated to be involved in herbicide detoxification, with the expression of its coding gene, *AtDTX21*, induced in *A. thaliana* seedlings via the application of the herbicide atrazine [69]. The overexpression of some MATE transporters in weeds results in herbicide resistance via a mechanism mediated through the root exudation of these xenobiotics [22].

Plant MATE proteins are also important in hormone regulation and homeostasis. The MATE transporter ADP1 has been demonstrated as a local regulator of auxin levels in meristematic tissues in *A. thaliana*, with mutations of ADP1 causing reduced plant growth [70]. Interestingly, some studies have shown an association between auxin levels and Al tolerance, such as occurs in *A. thaliana,* where AtDTX30 regulates both the auxin-mediated root development and hypersensitivity to Al via a mechanism involving citrate root exudation [71]. Similarly, in soybean, a stress leads to an accumulation of auxins at the root-tip level, with a decrease in the length of the primary roots and an increase in citrate exudation through the upregulation of GmMATE [72]. The levels of the phytohormone abscisic acid (ABA) are also partly regulated by MATE transporters, as demonstrated in *A. thaliana* for the role of DTX50, which regulates ABA efflux toward stomatal guard cells [33].

A direct role for MATE transporters as regulators of plant growth and development has been reported. In *A. thaliana,* the AtDTX MATE protein regulates root-hair growth [73], and LOC_Os12g36660 in *O. sativa* affects the grain weight [36]. The fertility of male gametophytes also depends on the MATE efflux carrier, as demonstrated for DTX34 in *A. thaliana* [74].

## 4. MATE Transporters in Plant–Pathogen Interactions

The mechanisms underlying plant–pathogen interactions are extraordinarily complex and multifaceted. On one hand, plants have evolved an extensive array of sophisticated strategies to recognize and mount effective defenses against potential pathogens [75]. These strategies include both preformed physical barriers and inducible biochemical responses, such as the activation of immune receptors, the production of antimicrobial compounds, and the reinforcement of cell walls [75]. On the other hand, phytopathogens have developed a complex arsenal of molecular and chemical tools to manipulate host cellular processes, suppress immune responses, and ultimately to overcome plant defenses [75]. These include the secretion of effector proteins that interfere with plant signaling pathways, the production of toxins that disrupt cellular integrity, and the deployment of enzymes that degrade cell walls. The dynamic interplay between these plant defenses and pathogen attack mechanisms determines the outcome of the interaction, which can range from successful pathogen invasion and disease development to effective plant resistance and pathogen suppression [75].

MATE transporters of both plants and pathogens are also reported to contribute to their interaction. In *A. thaliana*, the expression of the gene coding for the MATE protein EDS5 is induced by pathogens or abiotic stressors (e.g., UV-C light) [19]. EDS5 is essential in salicylic acid (SA) accumulation via the isochorismate synthase (ICS) pathway since it mediates the transport of isochorismate from the chloroplast to the cytoplasm [76,77]. Moreover, EDS5 is known to be involved in basal defenses against viruses, such as the yellow strain of *Cucumber mosaic virus* [CMV(Y)] and *Turnip crinkle virus* (TCV) [78]. Another MATE transporter of *A. thaliana*, named DTX18, has been demonstrated to allow for the extracellular accumulation of the antimicrobial compound coumaroylagmatine, which inhibits the germination of *Phytophthora infestans* spores but not the mycelial growth [79]. Transgenic potatoes expressing the *DTX18* gene were able to reduce the development of *P. infestans* spores, highlighting the importance of coumaroylagmatine in early-stage defenses [79]. In wheat (*Triticum aestivum*), the MATE transporter TaPIMA1 positively regulates defense mechanisms against *Rhizoctonia cerealis* by increasing the expression of defense-related genes, specifically of those coding for PR proteins [80]. The expression of the *TaPIMA1* gene was demonstrated to be induced by *R. cerealis*, as well as by exogenous H_2_O_2_ and jasmonic acid (JA), underlying the importance of this transporter in plant resistance against the sharp eyespot in wheat [80].

However, not all plant MATE transporters positively regulate plant defenses. Indeed, in *A. thaliana*, ADS1 was demonstrated to negatively affect plant disease resistance against *Pseudomonas syringae* pv. *tomato* DC3000 (*Pst* DC3000) [81]. Furthermore, Sun et al. (2011) suggested that ADS1 is a potential negative regulator in SA accumulation [81]. Similarly, the heterologous expression in *A. thaliana* of the genes *OsMATE1* and *OsMATE2*, coding in rice for two MATE transporters, resulted in the downregulation of some defense-related genes, such as those coding for PR proteins and β-1,3-Glucanase [82]. In addition, these transgenic plants exhibit a higher susceptibility to *Pst* DC3000 compared to wild-type plants [82].

Bacterial MATEs also have other important roles in the plant–pathogen interaction. The DinF transporter of *Ralstonia solanacearum* is involved in the efflux of some toxic compounds produced by *S. lycopersicum*. Mutations in the *dinF* gene lead to a reduced virulence of *R. solanacearum* when inoculated on tomato plants [83]. In *P. savastanoi* pv. *nerii*, a MATE transporter was demonstrated to modulate auxin homeostasis, particularly of IAA, during its interaction with its host *Nerium oleander* [84,85]. Indeed, through mutagenesis experiments, it was proved that the IAA efflux is mediated by the so-called Psn23 MatE transporter, which contributes to the maintenance of the optimal intracellular IAA concentrations during the infection process [85]. Similar results were also observed in *Pst* DC3000, where another MATE transporter was found to be important for bacterial fitness, colonization, and virulence in tomato plants [86].

MATE transporters are also implicated in the interaction between plants and their symbiotic microorganisms. As already mentioned, Mte1 is involved in ectomycorrhiza formation and morphogenesis between *T. vaccinum* and spruce through the transport of IAA [28]. In *Lupinus albus* L., the LaMATE2 protein has been demonstrated to be pivotal in nodule formation during *Rhizobium–Fabaceae* symbiotic interaction. Indeed, LaMATE2 mediates the release of the isoflavonoid genistein at the root level, especially under nitrogen deficiency and low phosphorous availability. The released genistein induces the expression of *NOD* genes in *Rhizobium* bacteria, leading to nodule formation [87]. In Cucurbitaceae, two MATE transporters (CmMATE1 and ClMATE1) have been reported to secrete the terpenoids cucurbitacin B and E in the rhizosphere. Moreover, it has been hypothesized that, in *Cucumis melo* L., the extrusion of the cucurbitacin B through CmMATE1 can increase the population of *Enterobacter* and *Bacillus* in the rhizosphere, conferring resistance to *Fusarium oxysporum* [88].

Recently, an increasing interest in the role of MATE transporters in plant–pathogen interaction has been observed. Unfortunately, most of these studies are currently restricted to in silico analyses, and, thus, they still need to be confirmed by experimental data. Concerning this, a potential role in defense mechanisms against *Xanthomonas citri* subsp. *citri* has been recently hypothesized for MATE transporters in *Citrus* spp., according to genomic and transcriptomic data [89]. Similarly, 66 MATE transporters were recently identified in apple, and they were indicated to be mainly involved in biotic stress responses [37].

The experimental evidence on the actual role of MATE proteins in plant–pathogen dialogue, as well as on the molecular mechanisms involved, are of paramount importance for the development of new sustainable and targeted strategies to control and manage the biotic diseases of plants. In Table 1, the main MATE transporters identified so far in microbes and plants are summarized, focusing on their substrates and on the physiological and biochemical processes in which these proteins are involved, including their role in plant–pathogen interactions.

## 5. Conclusions

Recently, there has been increasing interest in MATE transporters, as well as in the extremely different roles they play in all living organisms. However, up to now, very few MATE transporters have been fully characterized, both structurally and functionally. Currently, many studies are focused on plant MATEs, probably due to their multiple roles; however, the analysis of their expression under several stressors is still in its infancy. Conversely, MATE transporters of microorganisms account for a limited number of studies when compared to those conducted on plant MATEs. Many recent scientific papers are based on in silico analysis, and MATE proteins are identified and characterized mainly by comparative genomic data. Therefore, it is necessary to increase experimental studies to unveil the many different functions now hypothesized for these transporters. This information is pivotal to understanding many basic biological mechanisms, such as those involved in antibiotic resistance in important pathogenic bacteria for plants, animals, and humans.

The considerable growing interest related to these membrane efflux carriers is also associated with their potential as a target for several biotechnological approaches aiming to select plant varieties naturally more resistant to biotic and abiotic stressors, as well as their having a greater adaptability toward the most pressing challenges linked to ongoing global changes.

## Figures and Tables

**Table 1:** MATE transporters and their main substrates identified so far in microorganisms and plants.

	Organism	MATE Transporter	Substrates	Physiological and Biochemical Processes	References
Microorganisms	*Erwinia* *amylovora*	NorM	Norfloxacin, ethidium bromide, berberine	Resistance to biocides and antibiotics	[26]
	*Vibrio* *parahaemolyticus*	NorM	Norfloxacin, ciprofloxacin, ethidium, kanamycin, streptomycin	Resistance to biocides and antibiotics	[3]
	*Escherichia coli*	YdhE	Norfloxacin, ciprofloxacin, acriflavine, tetraphenylphosphonium ion	Resistance to antibiotics	[3]
	*Saccharomyces* *cerevisiae*	ERC1	Ethionine	Resistance to antimetabolite	[4,5]
	*Tricholoma* *vaccinum*	Mte1	Hygromycin B, Opus, indole-3-acetic acid	Resistance to antibiotics and fungicides, ectomycorrhiza formation	[27,28]
	*Ralstonia* *solanacearum*	DinF	Toxic compounds	Plant–pathogen interaction	[83]
	*Pseudomonas* *savastanoi pv. nerii*	MATE	Indole-3-acetic acid	Plant–pathogen interaction	[84,85]
	*Geotrichum* *citri-aurantii*	MATE	Guazatine	Resistance to fungicide	[29]
Plants	*Arabidopsis* *thaliana*	TT12	Glycosylated flavan-3-ol monomers	Transport of secondary metabolites	[44]
		FFT/DTX35	Anthocyanin	Transport of secondary metabolites	[45]
		AtMATE	Citrate	Al tolerance	[53]
		AtFRD3	Citrate	Fe homeostasis	[58]
		AtDTX1	Antibiotics, cadmium	Resistance to antibiotics, heavy metal tolerance	[62]
		AtDTX3	TNT	Bioremediation	[68]
		ADP1	Auxin	Auxin homeostasis Growth and development	[70]
		AtDTX21	Unknown	Atrazine detoxification	[69]
		AtDTX30	Auxin	Auxin homeostasis Root development Al tolerance	[71]
		EDS5	Isochorismate	Plant–pathogen interaction	[76]
		DTX18	Coumaroylagmatine	Plant–pathogen interaction	[79]
		DTX50	Abscisic acid	Growth regulation	[33]
	*Vitis vinifera*	AM1, AM3	Anthocyanin	Transport of secondary metabolites	[46]
		VvMATE38	Anthocyanin	Transport of secondary metabolites	[47]
	*Nicotiana tabacum*	NtMATE1, NtMATE2	Nicotine	Transport of secondary metabolites	[24]
		NtJAT1, NtJAT2	Nicotine	Transport of secondary metabolites	[49,50]
	*Populus trichocarpa*	PtrMATE1, PtrMATE2	Citrate	Al tolerance	[39]
	*Arachis hypogea*	AhFRDL1	Citrate	Al tolerance	[54]
	*Glycine max*	GmMATE13	Citrate	Al tolerance	[55]
		GmFRD3a, GmFRD3b	Citrate	Fe homeostasis	[59]
		GmMATE	Citrate	Al tolerance	[72]
	*Oryza sativa*	OsFRDL1	Citrate	Fe homeostasis	[60]
	*Lotus japonicus*	LjMATE	Citrate	Fe homeostasis	[38]
	*Lupinus albus*	LaMATE	Genistein	Symbiotic interaction	[87]
	*Cucumis melo*	CmMATE	Cucurbitacin B	Plant–microbiome interaction	[88]

## Data Availability

No new data were created or analyzed in this study; the data and the papers used to write this review are available on the Scopus database.
